# ﻿Two new species of *Tropodiaptomus* Kiefer, 1932 (Copepoda, Calanoida, Diaptomidae) from temporary waters in Thailand and Cambodia with a key to Southeast Asian species

**DOI:** 10.3897/zookeys.1249.157214

**Published:** 2025-08-25

**Authors:** Kamonwan Koompoot, Laorsri Sanoamuang

**Affiliations:** 1 Applied Taxonomic Research Center, Faculty of Science, Khon Kaen University, Khon Kaen 40002, Thailand Khon Kaen University Khon Kaen Thailand; 2 Laboratory of Biodiversity and Environmental Management, International College, Khon Kaen University, Khon Kaen 40002, Thailand Khon Kaen University Khon Kaen Thailand

**Keywords:** Biodiversity, endemic, freshwater, species-rich, taxonomy, *Tropodiaptomus kampucheaensis*, *Tropodiaptomus lannaensis*

## Abstract

Two new copepod species of the species-rich genus *Tropodiaptomus*, collected from temporary water habitats in Southeast Asia, are described. A critical morphological comparison has revealed that the two new species, *T. lannaensis***sp. nov.** from Thailand and *T. kampucheaensis***sp. nov.** from Cambodia, closely resemble their respective congeners, *T. ruttneri* (Brehm, 1923) and *T. doriai* (Richard, 1894). However, *T. lannaensis***sp. nov.** can be distinguished from other congeners, including *T. ruttneri*, by the following characters of the male P5: (1) the inner margin of the right basis has one small semicircular knob and one triangular knob; (2) the right second exopodal segment has a long, slender accessory spine inserted at the proximal third of the outer margin; and (3) the inner margin of the left exopod has a bilobed saw with a series of large denticles and smaller denticles near the distal end. *Tropodiaptomus kampucheaensis***sp. nov.** is different from all other species because it has (1) only one longitudinal hyaline lamella on the inner margin of the basis of the male right P5; (2) the one-lobed distal inner margin on the male left P5 exopod with uniform teeth; (3) the asymmetrical female genital-double somite (right margin expanded sub-proximally); and (4) the extraordinarily long inner seta on the P4 coxa of both sexes. The biogeography and keys to the species of *Tropodiaptomus* in Southeast Asia are presented.

## ﻿Introduction

With more than 75 species, the genus *Tropodiaptomus* Kiefer, 1932, is the most speciose of the diaptomid genera of freshwater calanoid copepods (Dussart and Defaye 2002; Walter and Boxshall 2025). Of these, up to 50 species are known in Africa, while the remaining species – aside from the Australian *Tropodiaptomus australis* Kiefer, 1936 – are found in Asia (Ranga Reddy 2013). In 1982, Kiefer made a taxonomic revision of *Tropodiaptomus* species known from Asian inland waters (Kiefer 1982). He also provided a key for male and female identification, which is documented for 15 species of the Asian taxa. Currently, the number of Asian species has been updated to 22, including three doubtful taxa (Ranga Reddy 2013; Defaye 2002; Saetang et al. 2020; Saetang and Maiphae 2023).

In Southeast Asia, four *Tropodiaptomus* species have been documented in the Philippines by Lai et al. (1979) and Lopez et al. (2017), including *T. australis* Kiefer, 1936, *T. gigantoviger* Brehm, 1933, *T. lanaonus* Kiefer, 1982, and *T. vicinus* (Kiefer, 1930). Five species have been recorded in Indonesia by Alekseev et al. (2013), namely *T. australis*, *T. doriai* (Richard, 1894), *T. hebereri* Kiefer, 1930, *T. vandouwei* (Fruhtl, 1924), and *T. vicinus*. In Malaysia and Singapore, three species [*T. hebereri*, *T. ruttneri* (Brehm, 1923), and *T. vicinus*] have been reported by Lim and Lai (2014). In Vietnam and Laos, four species [*T. foresti* Defaye, 2002, *T. oryzanus* Kiefer, 1937, *T. vicinus* (Kiefer, 1930), and *Tropodiaptomus* sp.] and one species [*T. vicinus* (Kiefer, 1930)] have been documented by Boonmak and Sanoamuang (2022) and Sivongxay (2005), respectively.

Recently, Sanoamuang and Dabseepai (2021) reported the existence of seven species of *Tropodiaptomus* in Thailand, including *T. hebereri*, *T. lanaonus*, *T. oryzanus* Kiefer, 1937, *T. ruttneri*, *T. vicinus*, *T. megahyaline* Saetang, Sanoamuang, & Maiphae, 2020, and *Tropodiaptomus* sp. In addition, Saetang and Maiphae (2023) described two more new species, namely *T. longiprocessus* Saetang & Maiphae, 2023, and *T. pedecrassum* Saetang & Maiphae, 2023. In Cambodia, only three species, *T. oryzanus*, *T. vicinus*, and *Tropodiaptomus* sp., have been identified by Chaicharoen and Sanoamuang (2022). While conducting extensive collections of diaptomid copepods from various freshwater habitats in Thailand and Cambodia, we encountered specimens of two previously undescribed *Tropodiaptomus* species. In this study, we describe and illustrate two new species: *T. lannaensis* sp. nov. and *T. kampucheaensis* sp. nov. Additionally, we provide a key to the *Tropodiaptomus* species of Southeast Asia and review their biogeography.

## ﻿Materials and methods

Samples were collected from a wide variety of freshwater habitats in Thailand in December 2012 and in Cambodia in June 2006, using a plankton net with a mesh size of 60 µm. The concentrated samples were then preserved in 70% ethanol or 4% formaldehyde immediately after collection. Specimens were dissected and mounted at 40–100× magnification under an Olympus SZ40 stereomicroscope. For illustrations, the habitus and all appendages were dissected and drawn at 400× and 1000× magnification with the aid of a drawing tube mounted to an Olympus CH30 compound microscope. The CorelDRAW Graphics Suite 2017 program was employed for the final version of the illustrated figures.

Specimens for scanning electron microscopy (SEM) were dehydrated in an ethanol series (50%, 70%, 80%, 90%, 95%, and 100%) for 15 minutes at each concentration. Specimens were dried in a critical-point dryer and mounted on stubs using adhesive tape under a stereomicroscope. The dried specimens were coated with gold in a sputter coater. The SEM photographs were taken using a scanning electron microscope (LEO, 1450VP).

The following abbreviations are used in both the text and the figures: **ae**, aesthetasc; **Enp**, endopod; **Exp**, exopod; **Exp-n**, exopodal segment n; **Enp-n**, endopodal segment n; **Pdg1–Pdg5**, pedigers 1–5; **P1–P5**, legs 1–5; **sp**, spine. The nomenclature and descriptive terminology follow Huys and Boxshall (1991), including the numbering of caudal setae (**I–VII**). Type specimens are deposited at the Thailand Natural History Museum (**THNHM**) and the Applied Taxonomic Research Center at Khon Kaen University, Thailand.

## ﻿Taxonomic section

### ﻿Infraclass Neocopepoda Huys & Boxshall, 1991

**Order Calanoida Sars, 1903**

**Family Diaptomidae Baird, 1850**

**Sub-family Diaptominae Kiefer, 1932**

**Genus *Tropodiaptomus* Kiefer, 1932**

#### ﻿ Tropodiaptomus lannaensis sp. nov.

Taxon classificationAnimaliaCalanoidaDiaptomidae

34FD9972-891B-582C-86F3-C68F4834969E

https://zoobank.org/A8B669D3-B5F5-4FC7-93E3-217F5C867BE0

Figs 2 , 3 , 4 , 5 , 6 , 7 , 8 , 9

Tropodiaptomus sp.: Sanoamuang and Dabseepai 2021: 5, 8, 16, 18, 23.

##### Material examined.

**Figure 1.**
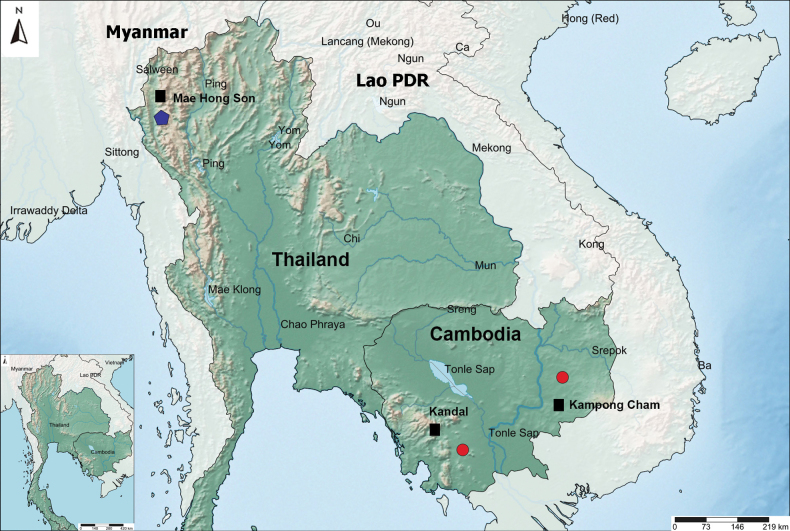
Sampling sites of *Tropodiaptomus lannaensis* sp. nov. in Thailand (blue pentagon) and *Tropodiaptomus kampucheaensis* sp. nov. in Cambodia (red spots). Black squares indicate cities.

***Holotype***: Thailand • one ♂ (adult), northern Thailand, Mae Hong Son Province, Mae La Noi District, 18°33'00.28"N, 97°91'01.42"E; 8 December 2012; water temperature 23.1 °C, pH 8.75, conductivity 386 µS cm^-1^, and dissolved oxygen 8.84 mg L^-1^. P. Dabseepai and K. Koompoot leg.; a temporary pond with aquatic plants near the road No. 108 (Fig. 1); accession number: THNHM-lv-18787; dissected, mounted on one slide in glycerol, covered with a coverslip, and sealed with nail polish. ***Paratypes***: Thailand • three ♂ (adult) and two ♀ (adult); date and collectors as for holotype; accession number: THNHM-lv-18789, undissected and preserved in 70% ethanol.

##### Description of adult male.

**Figure 2.**
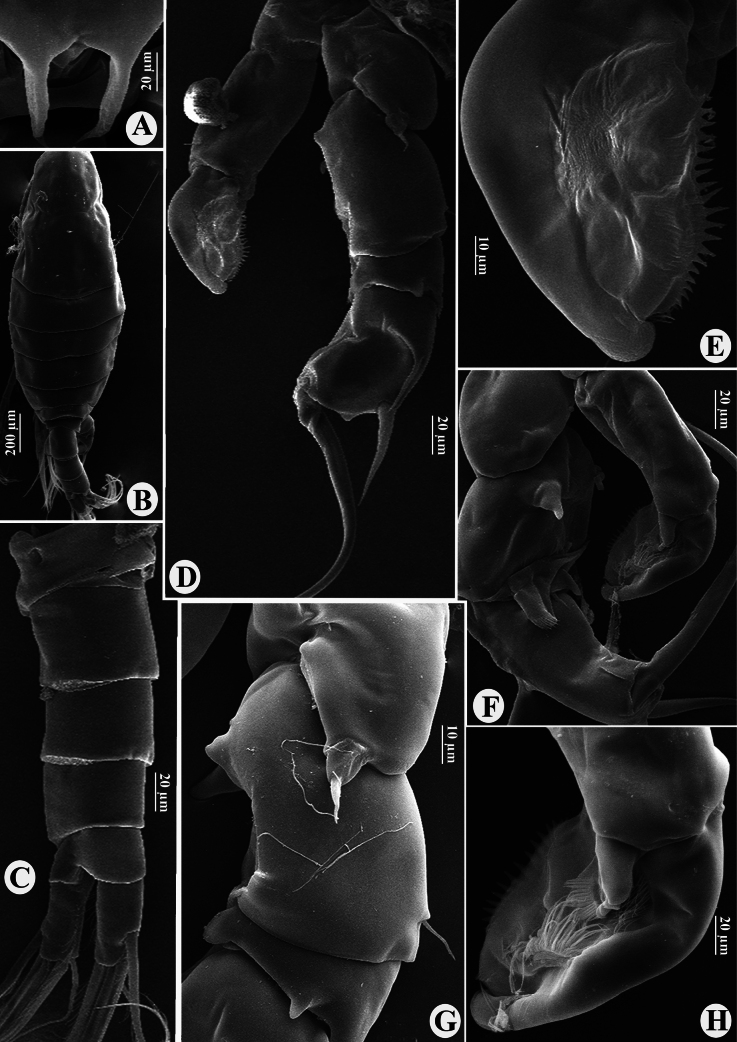
*Tropodiaptomus lannaensis* sp. nov., SEM photographs of male. A. Rostrum; B. Habitus, dorsal view; C. Genital somite, urosomites, and caudal rami, lateral view; D. P5, posterior view; E. Distal part of left P5, posterior view; F. P5, anterior view; G. Right P5 coxa, basis, and Exp-1, posterior view; H. Distal part of left P5, anterior view.

**Figure 3.**
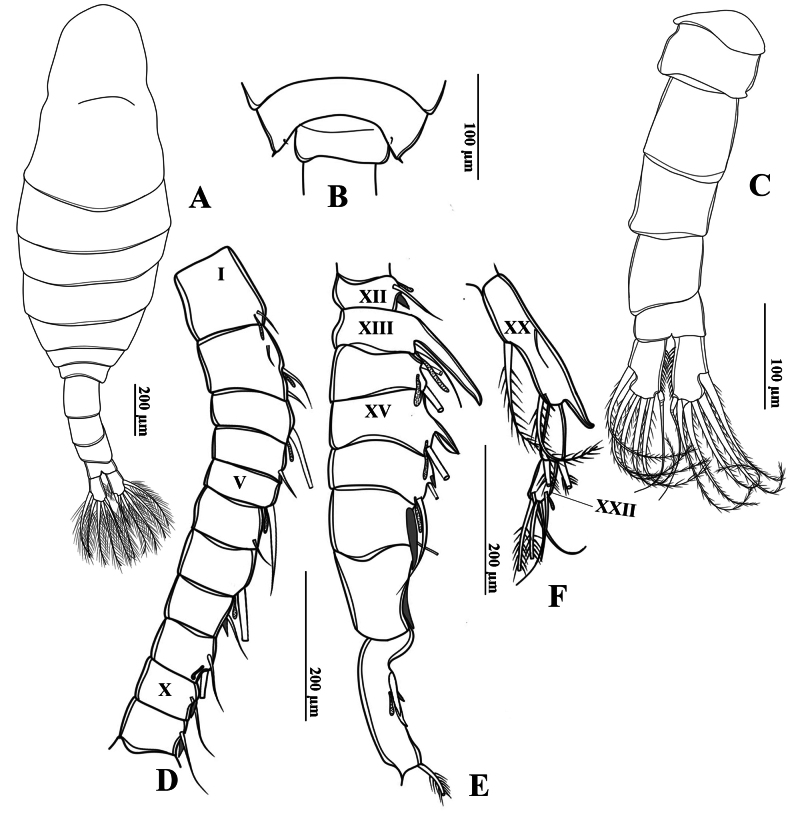
*Tropodiaptomus lannaensis* sp. nov., male. A. Habitus, dorsal view; B. Pediger 5 and genital somite, dorsal view; C. Urosome and caudal rami, ventral view; D–F. Right antennule; D. Segments 1–11; E. Segments 12–19; F. Segments 20–22.

Total body length, measured from anterior margin of rostrum to posterior margin of caudal rami, 1.16–1.21 mm (mean = 1.15 mm, *n* = 3) (Fig. 2B). Body smaller and slender than in female. Prosome ~ 2.2 × as long as urosome (Figs 2B, 3A). Rostrum (Fig. 2A) well-developed, with two spiniform processes. Pdg4 and Pdg5 separated by distinct septum dorso-laterally. Lateral wings of Pdg5 small and slightly asymmetrical; right wing larger than left one; both wings with one tiny spine at distal corner and one inner sensillum-like spine (Fig. 3B).

Urosome (Figs 2C, 3A–C) with five somites, unornamented. Genital somite slightly dilated postero-laterally on right side, shorter than wide. Urosomite 2 longer than wide, urosomite 3 approximately as long as wide, urosomite 4 with expanded right dorso-posterior corner (Fig. 3A). Urosomites 2 and 3 without ventral hairs (Figs 2C, 3C). Urosomites 3 and 4, anal somite, and caudal rami bent to right side. Anal somite asymmetrical, shorter than preceding urosomites. Caudal rami symmetrical, each ramus ~ 2.3 × as long as wide, inner margin hairy (Fig. 3C). Ventral surface of right caudal ramus without any prominence structures. Each ramus armed with six setae, subequal in length and size, plumose: dorsal seta bare and thinner than others.

**Figure 4.**
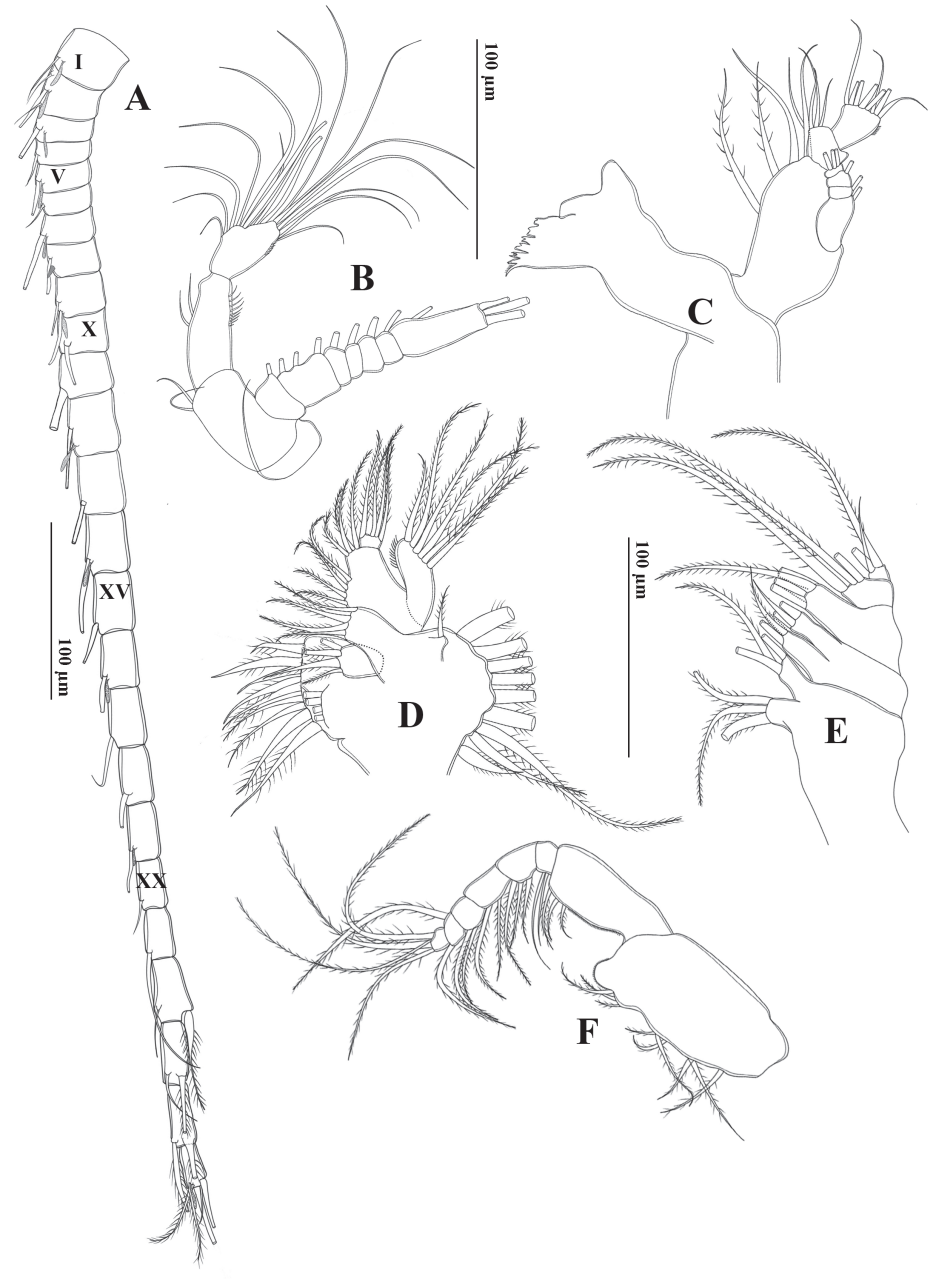
*Tropodiaptomus lannaensis*sp. nov., male. A. Left antennule; B. Antenna; C. Mandible; D. Maxillule; E. Maxilla; F. maxilliped.

Antennules asymmetrical, extending to approximately posterior end of genital somite. Left antennule (Fig. 4A): 25-segmented. Armature formulae as in Table 1. Right antennule geniculated (Fig. 3D–F), consisting of 22 segments, strongly dilated between segment XIII and segment XVIII (Fig. 3E). Segment XIII with largest strong spinous process. Antepenultimate segment (segment XX) longer than next segment. Spinous process on antepenultimate segment straight and slightly bent at distal end, reaching 2/3 of next segment (Fig. 3F). Armature formulae as in Table 2.

**Table 1.** Armature formulae of the left male antennule of *Tropodiaptomus lannaensis* sp. nov. The number of setae (Arabic numerals), aesthetascs (ae), and spines (sp) is given. The Roman numerals refer to segment numbers.

	Segment number												
	I	II	III	IV	V	VI	VII	VIII	IX	X	XI	XII	XIII
Number of elements	1+ae	3+ae	1+ae	1	1+ae	1	1+ae	1+sp	2+ae	1	1	1+ae+sp	1
	XIV	XV	XVI	XVII	XVIII	XIX	XX	XXI	XXII	XXIII	XXIV	XXV	
Number of elements	1+ae	1	1+ae	1	1	1+ae	1	1	2	2	2	5+ae	

**Table 2.** Armature formulae of the right male antennule of *Tropodiaptomus lannaensis*sp. nov. The number of setae (Arabic numerals), aesthetascs (ae), spines (sp), and spiniform processes (spr) is given. The Roman numerals refer to segment numbers.

	Segment number										
	I	II	III	IV	V	VI	VII	VIII	IX	X	XI
Number of elements	1+ae	3+ae	1+ae	1	1+ae	1	1+ae	1+sp	2+ae	1+sp	1+sp
	XII	XIII	XIV	XV	XVI	XVII	XVIII	XIX	XX	XXI	XXII
Number of elements	1+ae+sp	1+ae+spr	2+ae	2+ae+spr	2+ae+sp	1+sp	sp	1+ae +sp+2spr	4+spr	2	5+ae

Antenna (Fig. 4B) biramous. Coxa and basis with one and two bare setae on inner distal corner, respectively. Enp two-segmented; Enp-1 with two setae along inner margin and spinules on distal part of outer margin; Enp-2 with nine setae along inner margin, seven setae apically; all setae bare. Exp seven-segmented: Exp-1–6 with 1, 3, 1, 1, 1, 1, setae along inner margin; Exp-7 with one seta on inner margin and three setae apically; all setae bare.

Mandible (Fig. 4C): ~ 6 cuspidate teeth dorsally and one seta on coxal gnathobase dorsally. Basis with two serrate setae and two bare setae along inner margin. Enp-1 with four setae on inner distal corner. Enp-2 with eight setae apically. Exp four-segmented. Exp-1–3 each with one seta on inner margin; Exp-4 with three setae apically; all setae bare.

Maxillule (Fig. 4D): praecoxal arthrite with nine strong setae laterally and four slender submarginal setae. Coxal endite with four setae; coxal epipodite with nine setae. Basal endites fused to segment bearing them: proximal and distal endites, each with four setae apically; basal exite with one short seta. Enp-1 and Enp-2 each with four setae apically, proximal segment fused to basis. Exp unsegmented with six setae apically and hairy inner margin.

Maxilla (Fig. 4E): praecoxa fused to coxa. Proximal and distal endites on praecoxa, each with three setae apically. Two coxal endites, each with three setae apically. Allobasis with four setae apically. Enp two-segmented, each with three setae.

Maxilliped (Fig. 4F): four medial lobes on syncoxa: setal formulae 1, 2, 3, 3, respectively; subdistal inner margin produced into a spherical lobe with a patch of tiny spinules. Basis with three setae along distal inner margin, with a row of tiny spinules proximately. Enp six-segmented, with 2, 3, 2, 2, 2, and 4 setae, respectively.

**Figure 5.**
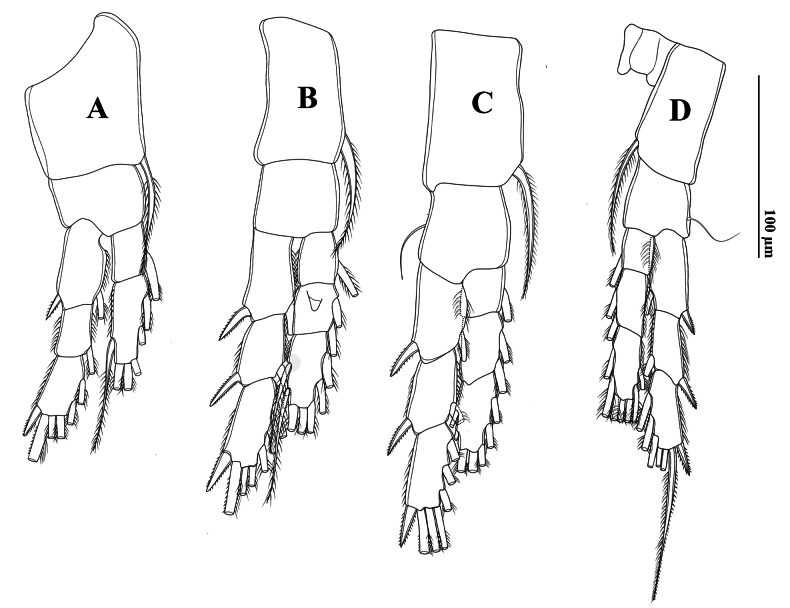
*Tropodiaptomus lannaensis*sp. nov., male. A. P1, posterior view; B. P2, posterior view; C. P3, posterior view; D. P4, posterior view.

Swimming legs (P1–P4) (Fig. 5A–D) biramous, with three-segmented rami, except for two-segmented Enp on P1. Exp longer than Enp. Each coxa on P1–P4 with one pinnate seta at innermost distal corner. P1–P3 bases without setae. P4 basis with one outer seta (Fig. 5D). P2 Enp-2 with Schmeil’s organ on posterior surface of (Fig. 5B). Armature formulae of P1–P4 as in Table 3.

**Table 3.** Armature formulae of the swimming legs of *Tropodiaptomus lannaensis*sp. nov. The number of setae (Arabic numerals) and spines (Roman numerals) is given in the following sequence: outer-inner margin or outer-apical-inner margin.

	Coxa	Basis	Exp			Enp		
			1	2	3	1	2	3
P1	0-1	0-0	I-1	0-1	I-3-2	0-1	1-2-3	–
P2	0-1	0-0	I-1	I-1	I-3-3	0-1	0-2	2-2-3
P3	0-1	1-0	I-1	I-1	I-3-3	0-1	0-2	2-2-3
P4	0-1	1-0	I-1	I-1	I-3-3	0-1	0-2	2-2-3

**Figure 6.**
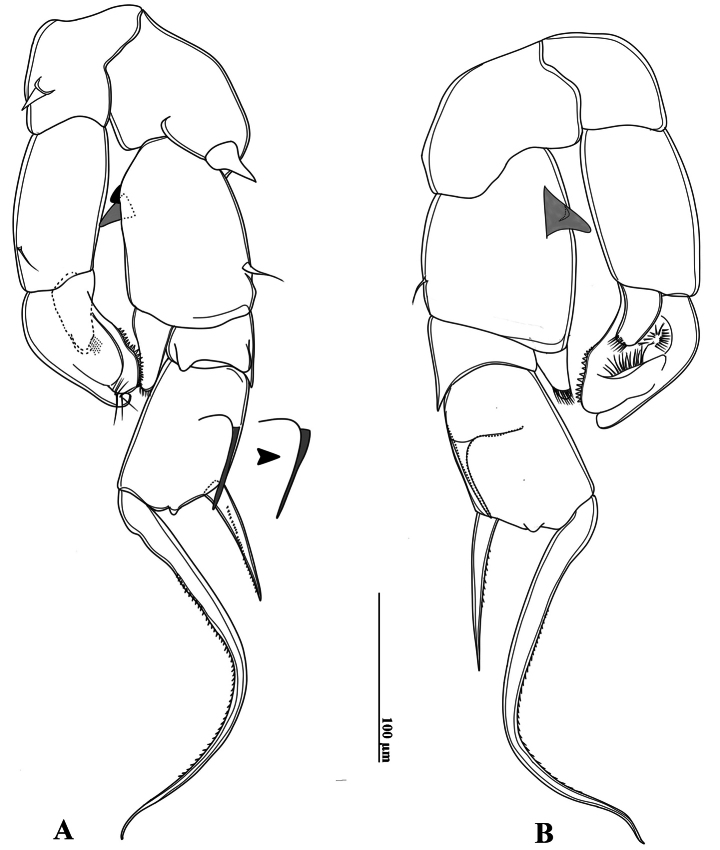
*Tropodiaptomus lannaensis*sp. nov., male. A. P5, posterior view; B. P5, anterior view.

P5 (Figs 2D–H, 6A, B): intercoxal sclerite fused to coxa, produced into a triangular lobe distally (Fig. 6A). Coxae asymmetrical, right part more prominent, each bears a stout spine on posterior surface; right spine larger than left spine. Right P5 (Figs 2D, 6A, B), basis rectangular, ~1.5× as long as wide; ornamented with a small semicircular knob at proximal inner margin and a larger, triangular hyaline knob occurring close to sub-proximal inner margin (Figs 2G, 6A); subdistal outer margin with a thin seta. Exp-1 shorter than wide, with a hyaline lobe on inner margin; outer distal corner with an acute tip (Fig. 6A, B). Exp-2 somewhat cylindrical, ~1.5× as long as wide, carrying accessory slender spine located at proximal 1/3 of outer margin and ~ 3/4 as long as outer distal spine (Figs 2D, 6A, B); outer distal spine moderately strong, slightly curved and acutely pointed, ornamented with spinules along inner margin, ~0.8 length of Exp-2, and located at anterior 2/3 of segment (Fig. 6A, B). Distal accessory element minute, situated close to insertion of end claw (Figs 2D, 6A). Enp one-segmented, conical shaped, reaching ~1/3 of Exp-2, bearing a row of apical spinules.

Left P5 (Figs 2D–F, H, 6A, B) slightly bent inwards, reaching to mid-length of right Exp-2. Coxa with relatively short spine inserted on ~ mid-length posterior lobe at outer margin. Basis rectangular, elongated, ~ 1.8 × as long as wide, bearing a small smooth seta at outer distal margin (Figs 2D, 6A); inner margin almost straight, without hyaline outgrowths. Exp somewhat semicircular-shaped, flattened and ~ 1.6 × as long as wide; outer margin curved, inner margin two-lobed, both lobes with large denticles, small denticles near distal end only (Fig. 2E, H); apex of Exp with typical digitiform appendix and spinulate seta, while anterior surface displays two elongate, hairy pads (Fig. 2E, H). Enp one-segmented and dilated proximally, extending to mid-length of Exp; apex rounded with a row of subapical spinules (Fig. 6A, B).

##### Description of adult female.

**Figure 7.**
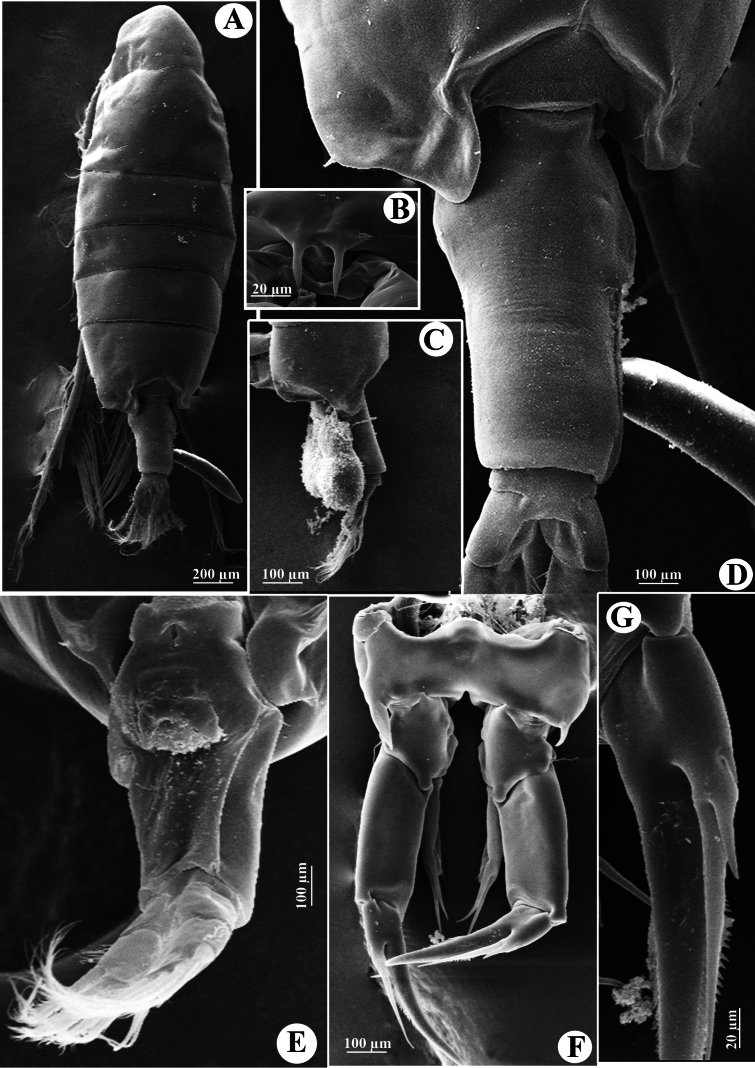
*Tropodiaptomus lannaensis*sp. nov., SEM photographs of female. A. Habitus, dorsal view; B. Rostrum; C. Last pedigerous somite and urosome, lateral view; D. Last pedigerous somite and urosome, dorsal view; E. Urosome, ventral view; F. P5, anterior view; G. P5Exp-2–3.

**Figure 8.**
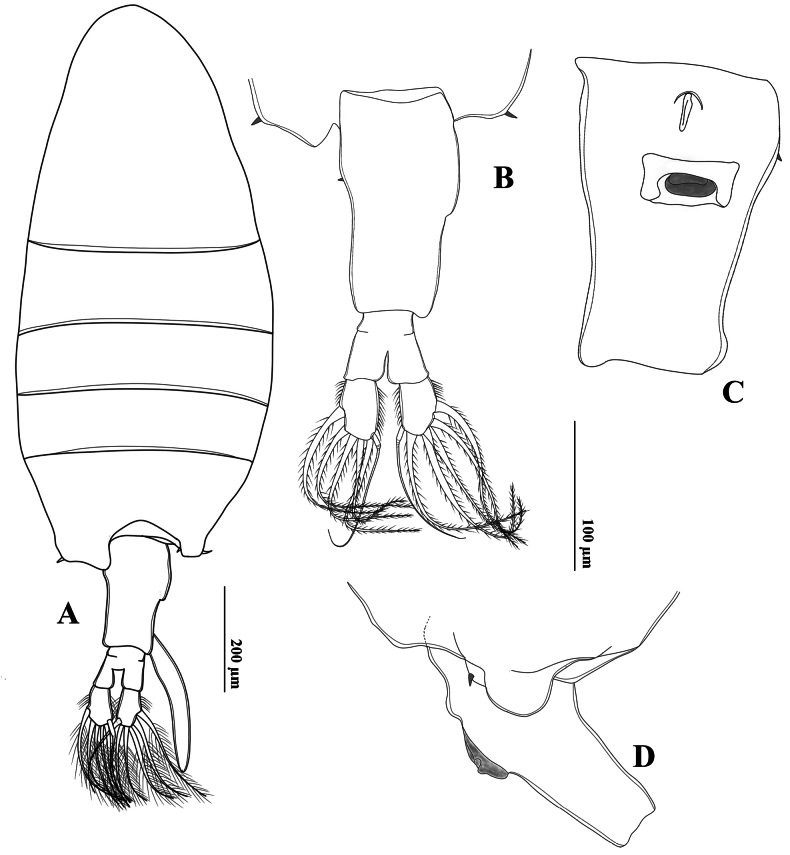
*Tropodiaptomus lannaensis*sp. nov., female. A. Habitus, dorsal view; B. Last pedigerous somite, urosome, and caudal rami, dorsal view; C. Urosome, ventral view; D. Last pedigerous somite and urosome, lateral view.

Total body length, measured from anterior margin of rostrum to posterior margin of caudal rami, 1.27–1.41 mm (mean 1.35 mm, *n* = 3) (Figs 7A, 8A). Prosome: urosome ratio ~ 2.1: 1. Rostrum (Fig. 7B) symmetrical, with moderately long point and ornamented with a pair of sensilla. Pdg4 and Pdg5 completely fused. Last pedigerous somite with asymmetrical posterolateral wings (Figs 7D, 8A, B); left wing larger and longer than right wing. Each wing with one hyaline spine (Figs 7D, 8B, D).

Urosome (Figs 7D, E, 8A–D) two-segmented. Genital double-somite (Figs 7D, 8A, B) asymmetrical, longer than urosomite 2, anal somite, and caudal rami combined (Fig. 8A, B); right proximal region slightly expanded, right distal corner produced into a small lobe; left margin slightly curved, with a small spine at proximal 1/3 of somite (Fig. 8B). A pair of gonopores and copulatory pores located centrally at ~ 1/3 length of genital double-somite (Figs 7E, 8C). Urosomite 2 symmetrical, shorter than wide. Anal somite symmetrical, as long as length of caudal rami (Fig. 8B). Caudal rami parallel, symmetrical; each ramus ~ 1.5 × as long as wide and with hairy outer and inner margins (Fig. 8A, B). All principal caudal setae slightly dilated anteriorly.

Left antennule, antenna, mouthparts, and P1–P4 similar to those of the male. Antennules symmetrical.

**Figure 9.**
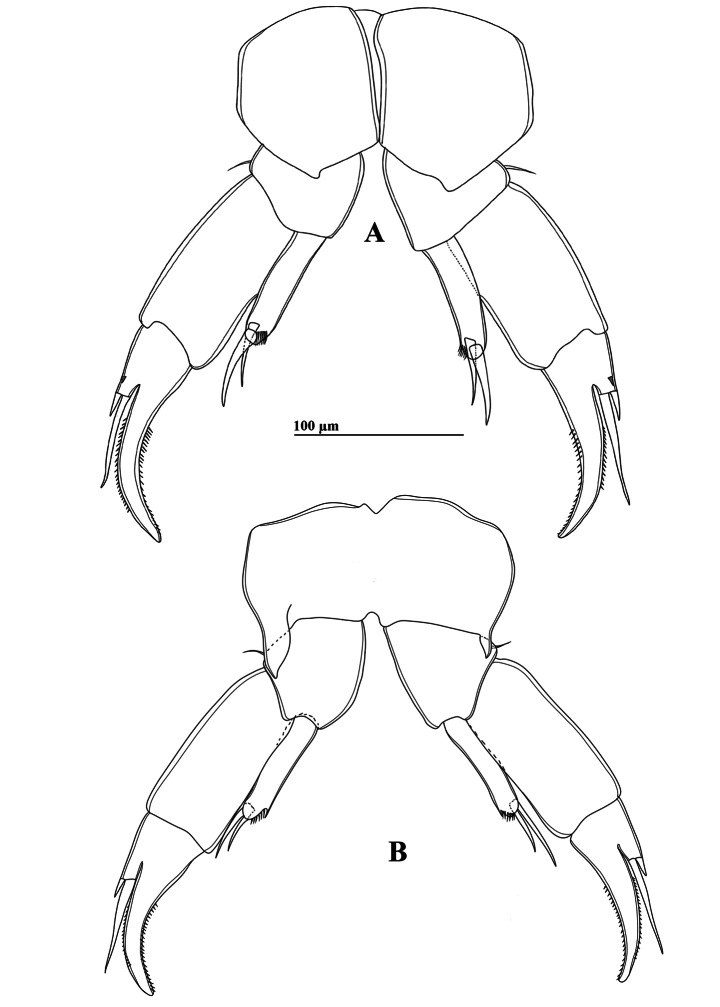
*Tropodiaptomus lannaensis*sp. nov., female. A. P5, posterior view; B. P5, anterior view.

P5 (Figs 7F, G, 9A, B) asymmetrical. Intercoxal sclerite forming a narrow, elongate triangle. Coxa massive, with broadly strong process situated anterolaterally on distal outer margin; coxal spine on right side larger and longer than left side (Figs 7F, 9B). Basis with a bare, minute sensory seta on outer margin (Figs 7F, 9B). Exp (Figs 7F, G, 9A, B) three-segmented. Exp-1 cylindrical, ~ 1.9 × as long as wide, convex outer margin and almost straight inner margin; Exp-2 sub-triangular, with a row of strong spinules along both margins; Exp-3 fused into small prominence on Exp-2, armed with one short spine and one long seta apically. Enp one-segmented (Figs 7F, 9A, B), ~ 0.7 × as long as Exp-1, armed with two strong, unequal spiniform setae; outer seta longest, with a row of apical spinules.

##### Distribution and ecology.

*Tropodiaptomus lannaensis*sp. nov. has so far been found exclusively in its type locality, a temporary pond in Mae Hong Son Province, northern Thailand. At the time of the new species’ collection, no other calanoid copepods were present at the same site. Representatives of the new species were recorded solely once among more than 5,000 sampled locations in Thailand. This species is rare and currently endemic to Thailand.

##### Etymology.

The specific epithet *lannaensis* is derived from Lanna, the name of the area in present-day northern Thailand from the 13^th^ to 18^th^ centuries in which the type locality is located. The name is an adjective in the nominative singular, feminine gender. The suffix -*ensis* originates from Latin and indicates the specific origin.

#### ﻿ Tropodiaptomus kampucheaensis sp. nov.

Taxon classificationAnimaliaCalanoidaDiaptomidae

5433833C-8EC5-564D-9B93-5F38877B5AB0

https://zoobank.org/92E502B1-A9FB-430E-85C8-3B3DB00DB63A

**Figure 10.**
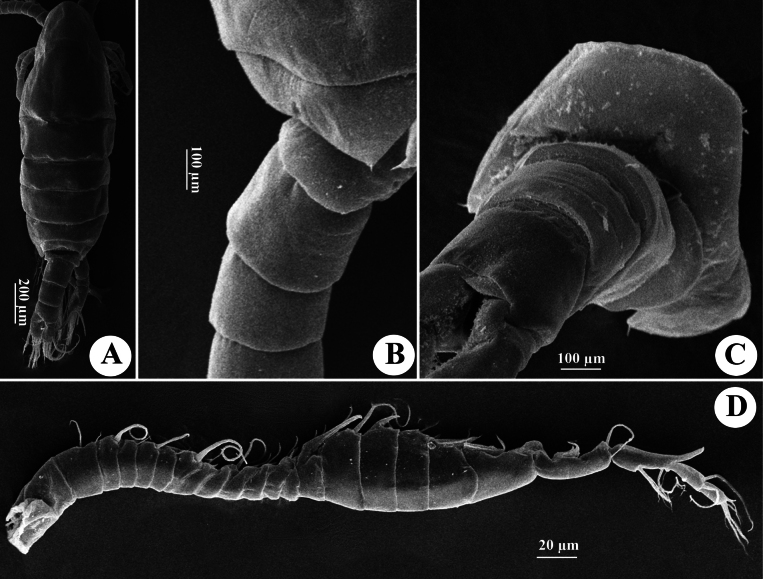
*Tropodiaptomus kampucheaensis* sp. nov., SEM photographs of male. A. Habitus, dorsal view; B. Pedigers 4 and 5, genital somite, and urosomites 2–4, lateral view; C. Last pedigerous somite and urosome, dorsolateral view; D. Right antennule.

**Figure 11.**
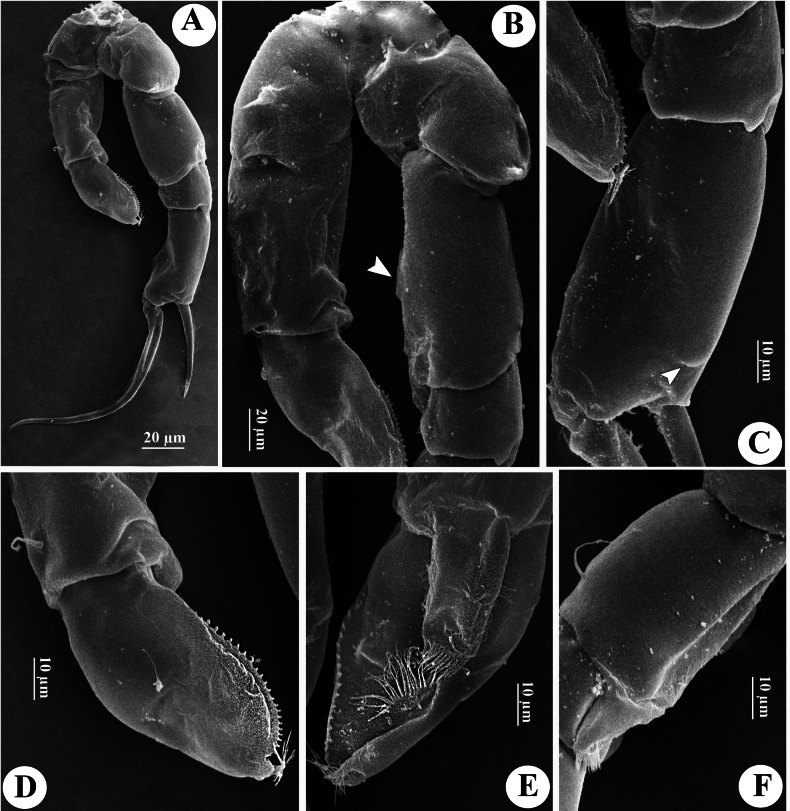
*Tropodiaptomus kampucheaensis* sp. nov., SEM photographs of male. A. P5, posterior view; B. Proximal part of P5, posterior view (white arrow points to hyaline membrane on inner margin of right basis); C. Right P5Exp-1–2 (white arrow points to a crescent-shaped lamella); D. Distal part of left P5, posterior view; E. distal part of left P5, anterior view; F. Right P5 basis and Enp, anterior view.

**Figure 12.**
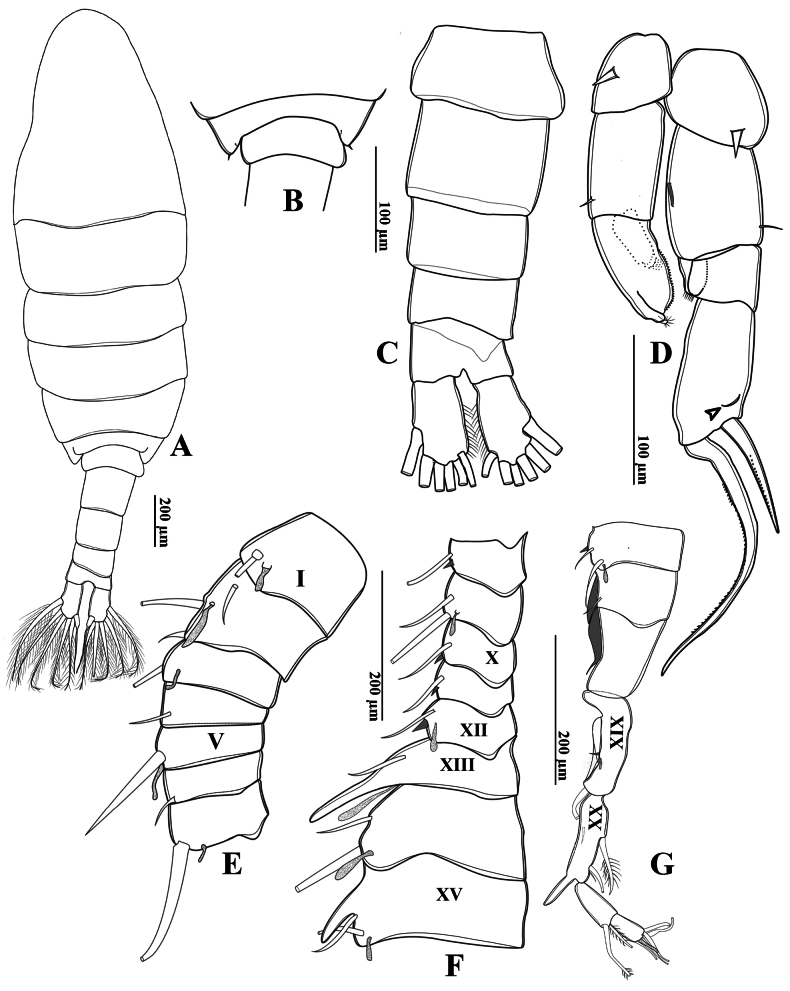
*Tropodiaptomus kampucheaensis* sp. nov., male. A. Habitus, dorsal view; B. Pediger 5 and genital somite, dorsal view; C. Urosome and caudal rami, ventral view; D. P5, posterior view (arrow points to a crescent-shaped lamella); E–G. Right antennule; E. Segments 1–7; F. Segments 8–15; G. segments 16–22.

**Figure 13.**
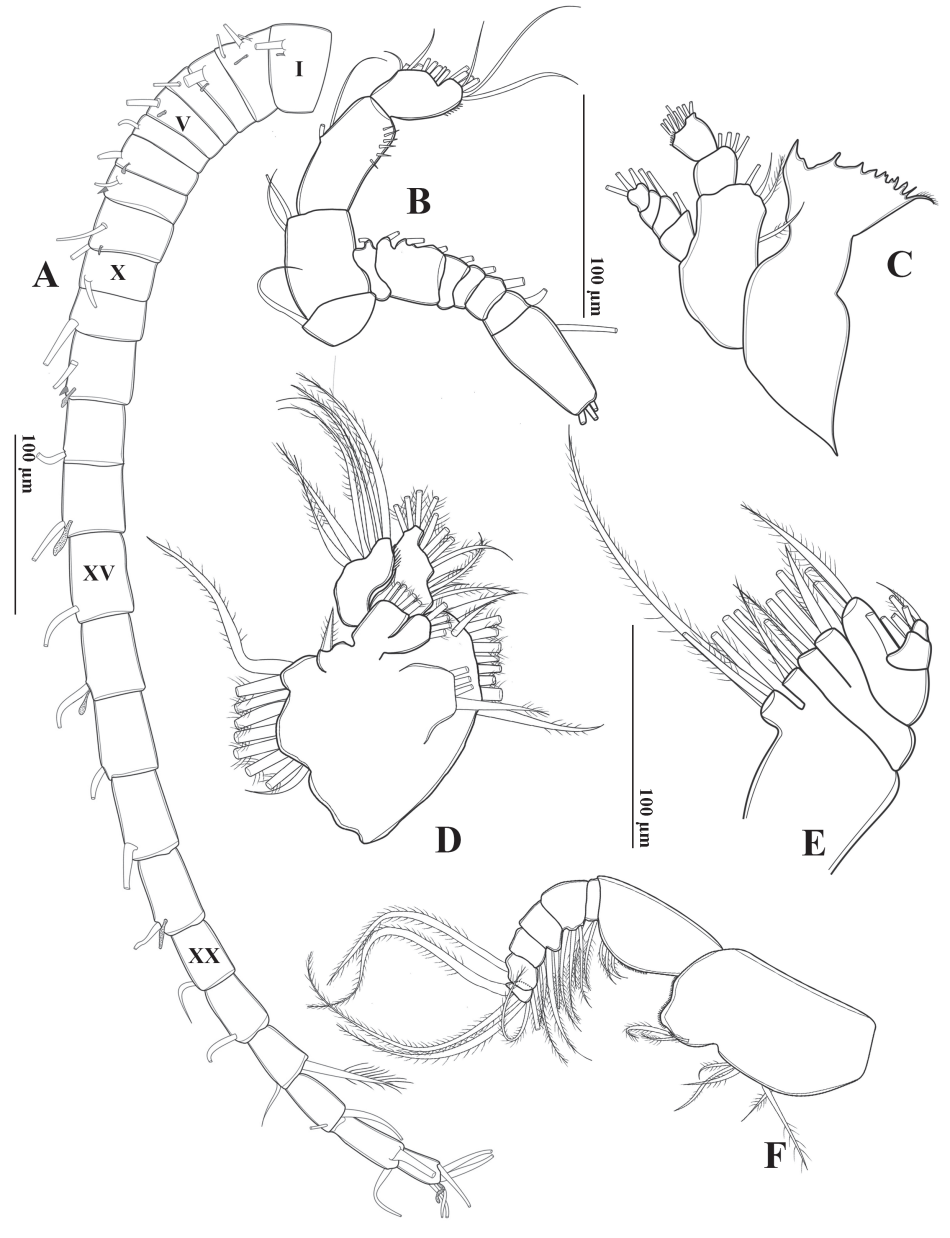
*Tropodiaptomus kampucheaensis* sp. nov., male. A. Left antennule; B. Antenna; C. Mandible; D. Maxillule; E. Maxilla; F. Maxilliped.

**Figure 14.**
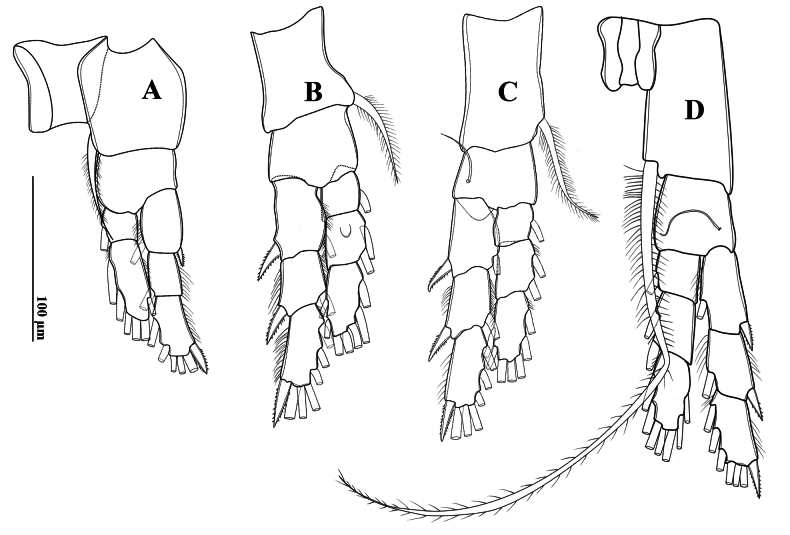
*Tropodiaptomus kampucheaensis* sp. nov., male. A. Posterior view; B. P2, posterior view; C. P3, posterior view; D. P4, posterior view.

**Figure 15.**
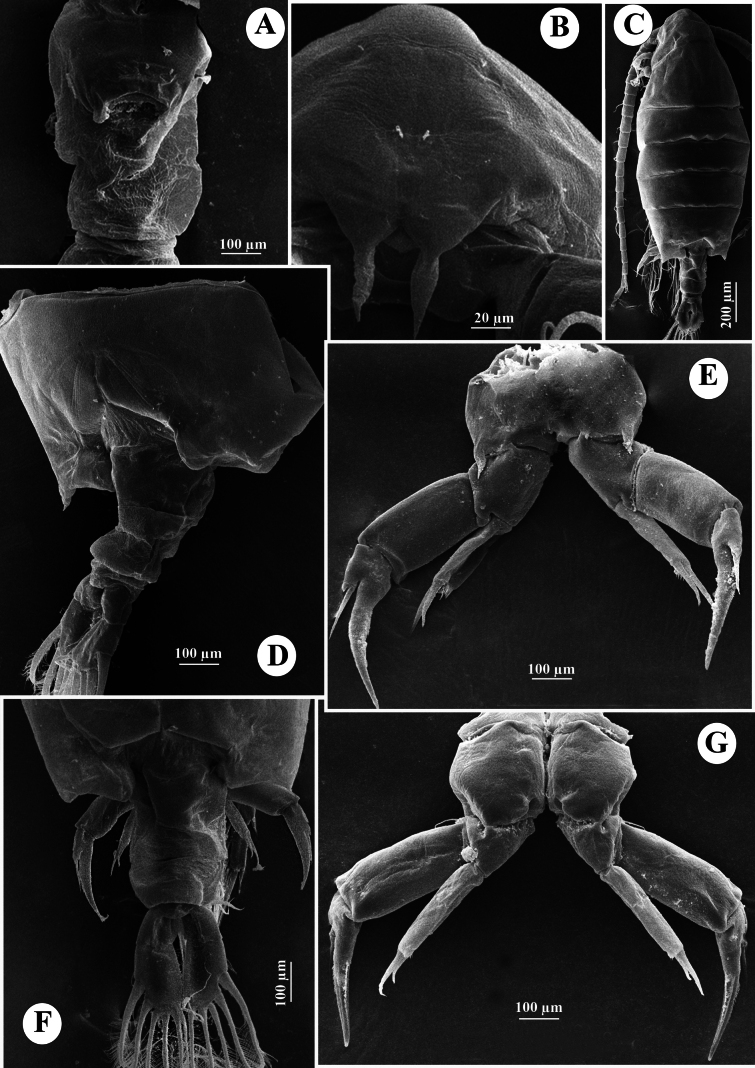
*Tropodiaptomus kampucheaensis* sp. nov., SEM photographs of female. A. Genital double-somite, ventral view; B. Rostrum; C. Habitus, dorsal view; D. Last pedigerous somite and urosome, dorsolateral view; E. P5, anterior view; F. Last pedigerous somite and urosome, dorsal view; G. P5, posterior view.

**Figure 16.**
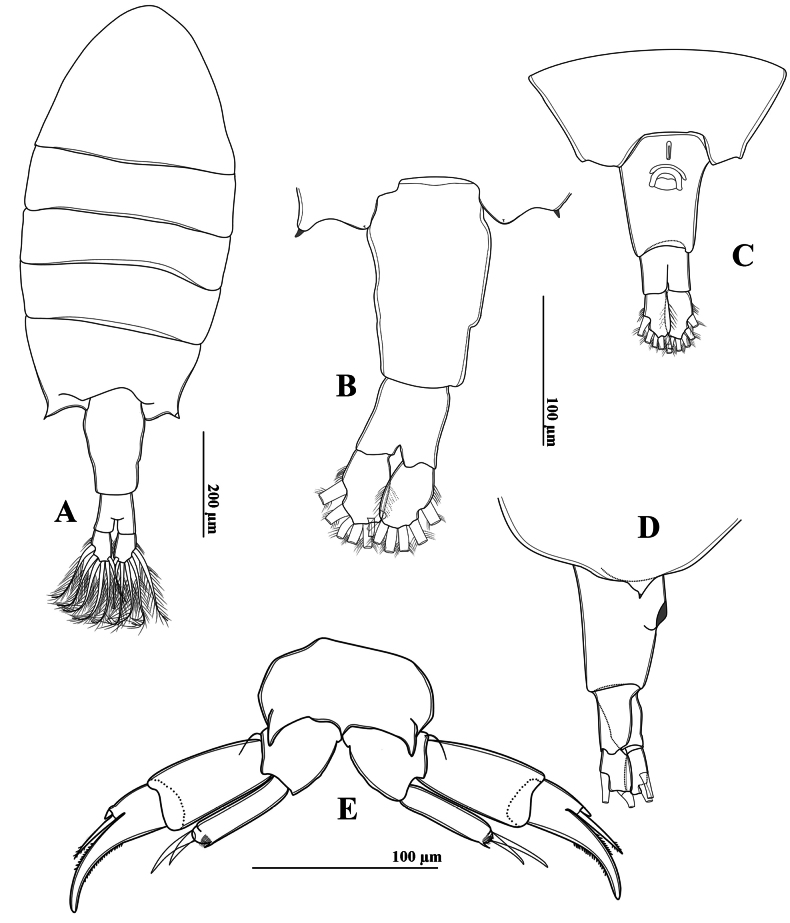
*Tropodiaptomus kampucheaensis* sp. nov., female. A. Habitus, dorsal view; B. Last pedigerous somite, urosome, and caudal rami, dorsal view; C. Last pedigerous somite and urosome, ventral view; D. Last pedigerous somite and urosome, lateral view; E. P5, anterior view.

Figs 10 , 11 , 12 , 13 , 14 , 15 , 16

Tropodiaptomus sp.: Chaicharoen and Sanoamuang (2022): 1, 6, 7, 9, 11, 12, 13.

##### Material examined.

***Holotype***: Cambodia • one ♂ (adult), Cambodia, Kampong Cham Province, Tbong Khmum District, 12°32'34"N, 105°36'94"E; 16 June 2006; water temperature 28.9 °C, pH 7.7, and conductivity 108.6 µS cm^-1^. W. Mahasrap leg.; a rice field (Fig. 1); accession number: THNHM-lv-19360; dissected, mounted on one slide in glycerol, covered with a coverslip, and sealed with nail polish. ***Allotype***: Cambodia • one ♀ (adult); location, date and collectors as for holotype; accession number: THNHM-lv-19361, completely dissected, mounted on one slide in glycerol, covered with a coverslip, and sealed with nail polish. ***Paratypes***: Cambodia • three ♂ (adult) and three ♀ (adult); date and collectors as for holotype; accession number: THNHM-lv-19362, undissected and preserved in 70% ethanol.

##### Additional occurrence locality.

A temporary roadside canal, Kandal province, Cambodia (11°38'72"N, 104°20'19"E); 16 June 2006; water temperature 29.2 °C, pH 8.2, and conductivity 94.1 µS cm^-1^.

##### Description of adult male.

Total body length, measured from anterior margin of rostrum to posterior margin of caudal rami, 0.94–0.95 mm (mean 0.947 mm, *n* = 3) (Figs 10A, 12A). Body smaller and slender than in female. Prosome ~ 2.5 × as long as urosome (Fig. 10A, 12A). Pdg4 separated dorso-laterally from Pdg5. Lateral wings of Pdg5 small, symmetrical, with one tiny spine at distal corner and one inner sensillum-like spine (Figs 10C, 12B).

Urosome (Figs 10A–C, 12 A–C) with five somites, unornamented. Genital somite dilated postero-laterally on both sides (Fig. 12C), shorter than wide. Urosomite 2 ~ as long as wide, urosomites 3 and 4 shorter than wide. Urosomites 2 and 3 without hairs on ventral side (Figs 10B, 12C). Urosomite 4 with expanded right dorso-posterior margin. Anal somite asymmetrical, left side slightly longer than right side. Caudal rami symmetrical, each ramus ~ 1.5 × as long as wide, inner margins hairy (Fig. 12C). Ventral surfaces of both caudal rami without any prominent structures. Each ramus armed with five plumose setae and one base dorsal seta.

Antennules: asymmetrical, long, reaching to posterior end of genital somite. Left antennule (Fig. 13A): 25-segmented. Armature formulae as in Table 4. Right antennule geniculated (Figs 10D, 12E–G), consisting of 22 segments, strongly dilated between segment XIII and segment XVIII. Segment XIII with largest strong spinous process, one seta, and one aesthetasc. Antepenultimate segment (segment XX) longer than next segment. Spinous process on antepenultimate segment straight with an outwardly curved tip, reaching 2/3 of next segment (Figs 10D, 12G). Armature formulae as in Table 5.

**Table 4.** Armature formulae of the left male antennule of *Tropodiaptomus kampucheaensis* sp. nov. The number of setae (Arabic numerals), aesthetascs (ae), and spines (sp) is given. The Roman numerals refer to segment numbers.

	Segment number												
	I	II	III	IV	V	VI	VII	VIII	IX	X	XI	XII	XIII
Number of elements	1+ae	3+ae	1+ae	1	1+ae	1	1+ae	1+sp	2+ae	1	1	1+ae+sp	1
	XIV	XV	XVI	XVII	XVIII	XIX	XX	XXI	XXII	XXIII	XXIV	XXV	
Number of elements	1+ae	1	1+ae	1	1	1+ae	1	1	2	2	2	5+ae	

**Table 5.** Armature formulae of the right male antennule of *Tropodiaptomus kampucheaensis*sp. nov. The number of setae (Arabic numerals), aesthetascs (ae), spines (sp), and spiniform processes (spr) is given. The Roman numerals refer to segment numbers.

	Segment number										
	I	II	III	IV	V	VI	VII	VIII	IX	X	XI
Number of elements	1+ae	3+ae	1+ae	1	1+ae	1	1+ae	1+sp	2+ae	1+sp	1+sp
	XII	XIII	XIV	XV	XVI	XVII	XVIII	XIX	XX	XXI	XXII
Number of elements	1+ae+sp	1+ae+spr	2+ae	2+ae+spr	2+ae+sp	1+sp	sp	1+ae+sp+2spr	4+spr	2	5+ae

Antenna (Fig. 13B) biramous. Coxa and basis with one and two bare setae on distal corner, respectively. Enp two-segmented; Enp-1 with two inner median setae and small spinules on distal outer margin; Enp-2 bilobed, bearing eight setae on inner lobe and six setae on outer lobe. Exp seven-segmented, longer than Enp. Exp 1–6 with setal formulae 1, 3, 1, 1, 1, and 1, respectively. Exp 7 with one inner seta and three apical setae.

Mandible (Fig. 13C): coxal gnathobase with cutting edge bearing eight well-chitinized teeth and one setulose seta. Basis with four inner setae; one located proximally and three distally. Enp two-segmented; Enp-1 with four setae on inner distal corner; Enp-2 with eight setae apically. Exp four-segmented; Exp-1–3 each with one seta on inner margin; Exp-4 with three setae apically; all setae bare.

Maxillule (Fig. 13D): precoxal arthrite with nine strong setae laterally, four slender submarginal setae, and one anterior seta. Coxal epipodite with nine setae; coxal endite with four setae. Basal endites each with four setae proximally and four setae distally; basal exite with one outer seta. Enp with eight setae distally. Exp with six setae.

Maxilla (Fig. 13E): praecoxa fused to coxa. Proximal and distal endites on praecoxa each with three setae apically. Two coxal endites each with three setae apically. Allobasis with three setae apically. Enp two-segmented, each with three setae.

Maxilliped (Fig. 13F) praecoxa and coxa fused, three medial lobes on syncoxa: setal formulae 2, 3, 3, respectively; subdistal inner margin produced into a spherical lobe with a patch of tiny spinules. Basis with three setae at distal inner margin, with a row of tiny spinules proximately. Enp six-segmented, with 2, 3, 2, 2, 1+1, and 4 setae, respectively.

Swimming legs (P1–P4) (Fig. 14A–D) biramous, with three-segmented rami, except for two-segmented Enp on P1. Exp longer than Enp. Each coxa on P1–P4 with one pinnate seta at innermost distal corner. P4 coxa with an extraordinarily long inner seta (Fig. 14D). P1 and P2 bases without setae. P3 and P4 bases with a bare outer seta. P2 Enp-2 with a Schmeil’s organ on posterior surface (Fig. 14B). Armature formulae of P1–P4 as in Table 6.

**Table 6.** Armature formulae of the swimming legs (P1–P4) of *Tropodiaptomus kampucheaensis* sp. nov. The number of setae (Arabic numerals) and spines (Roman numerals) is given in the following sequence: outer-inner margin or outer-apical-inner margin.

	Coxa	Basis	Exp			Enp		
			1	2	3	1	2	3
P1	0-1	0-0	I-1	0-1	I-3-2	0-1	1-2-3	–
P2	0-1	0-0	I-1	I-1	I-3-3	0-1	0-2	2-2-3
P3	0-1	1-0	I-1	I-1	I-3-3	0-1	0-2	2-2-3
P4	0-1	1-0	I-1	I-1	I-3-3	0-1	0-2	2-2-3

P5 (Figs 11A–F, 12D) highly asymmetrical. Intercoxal sclerite reduced, inner distal margin not produced. Coxae fused to intercoxal sclerite. Right P5 (Figs 11A–C, F, 12D): right coxa larger than left one, with a triangular spine on outer subdistal corner of posterior surface. Basis (Figs 11 A, B, F, 12D) rectangular, ~ 1.5 × as long as wide, ornamented with a thin, longitudinal hyaline membrane located at mid-length of inner margin (Figs 11B, 12D), and a short seta at subdistal outer corner. Exp-1 (Figs 11A–C, 12D) shorter than wide, produce into acute process on outer distal corner. Exp-2 (Figs 11A, C, 12D) elongated, somewhat rectangular, ~ 1.9 × as long as wide, with a thin crescent-shaped lamella near base of principal lateral spine (Figs 11C, 12D). Principal lateral spine robust, slightly curved, acutely pointed, ~ 0.9 × as long as Exp-2 segment, located sub-distally, close to end claw, with spinules on distal outer and inner margins. End claw long, slender, and sickle-shaped, with a pointed tip and ornamented with spinules on inner margin; ~ 1.6 × as long as Exp-2. Enp one-segmented, conical, reaching of Exp-1, and bearing a row of apical spinules.

Left P5 (Figs 11A, B, D, E, 12D) slightly bent inwards, reaching to 1/4 of right Exp-2. Coxa as long as wide, with one triangular spine inserted on posterior lobe, at mid-distal outer margin. Basis (Figs 11B, 12D) rectangular, elongated, ~ 1.6 × as long as wide, with one short seta near outer corner distally; inner margin straight, without hyaline lamella. Exp (Fig. 11D, E) flattened, elongated, ~ 1.0 × as long as basis; inner margin one-lobed, with uniform serration; anterior surface ornamented with two hairy pads in center, proximal one located under Enp; apex of Exp with usual combination of digitiform appendix and spinulate seta. Enp (Fig. 11E) one-segmented, slightly cylindrical, extending to mid-length of Exp; apex rounded with a row of subapical spinules.

##### Description of adult female.

Total body length, measured from anterior margin of rostrum to posterior margin of caudal rami, 1.53–1.55 mm (mean 1.54 mm, *n* = 3). Prosome: urosome ratio ~ 2.3:1. Prosome similar to that of male. Rostrum (Fig. 15B) symmetrical, with moderate strong and acutely pointed, paired filaments. Pdg4 and Pdg5 completely fused. Last pedigerous somite (Figs 15D, F, 16B, C) with nearly symmetrical postero-lateral wings, each wing armed with one postero-lateral spine.

Urosome (Figs 15D, F, 16A–D) two-segmented. Genital-double somite (Figs 15A, D, F, 16B–D) asymmetrical, ~ twice as long as wide, longer than anal somite and caudal rami combined; left proximal margin slightly dilated, with ~2/3 of right proximal margin expanded; distal part narrower than proximal part. Genital area on ventral surface with opercular pad protecting gonopores, and with rectangular and semicircular expansion (Fig. 16C). Anal somite ~ 1.2 × as long as caudal rami. Caudal rami (Figs 15F, 16B) symmetrical, ~ 2.1 × as long as wide, parallel, with hairy outer and inner margins, each ramus with six setae; dorsal setae jointed, longer than principal setae.

Left antennule, antenna, mandible, maxillule, maxilla, maxilliped, and P1–P4 similar to those of the male.

P5 (Figs 15E, G, 16E) symmetrical. Coxa roughly spherical, with a stout, triangular spine anteriolaterally. Basis (Fig. 16E) with one short, smooth sensory seta on distolateral margin (Fig. 16E). Exp (Figs 15E, G, 16E) three-segmented; Exp-1 cylindrical, ~ 2 × as long as wide, with almost straight inner and outer margins. Exp-2 sub-triangular, with a row of strong spinules along both margins; Exp-3 fused into a small prominence on Exp-2, armed with one short spine and one long seta apically. Enp (Fig. 16E) one-segmented, ~ 0.8 × as long as Exp-1, armed with two strong, unequal spiniform setae; outer seta longest, with a row of tiny spinules apically.

##### Distribution and ecology.

Currently, *T. kampucheaensis* sp. nov. has been found only in two temporary-water habitats: a temporary pond in Kampong Cham Province and a shallow canal in Kandal Province, Cambodia. Representatives of the new species were found in two sites from the 147 sampled locations in five provinces (Kampong Chnang, Kampong Cham, Kendal, Prey Veng, and Takeo) in Cambodia. This species is rare and currently endemic to Cambodia. The newly discovered species was observed alongside two diaptomid species, namely *Allodiaptomus raoi* Kiefer, 1936, and *Mongolodiaptomus mekongensis* Sanoamuang & Watiroyram, 2018.

##### Etymology.

The specific name *kampucheaensis* refers to the name Kampuchea, which is the native name for Cambodia in the Khmer language, where the type locality is located. The suffix -*ensis* originates from Latin and indicates the specific origin.

### ﻿Keys to Southeast Asian species of the genus *Tropodiaptomus* Kiefer, 1932

Males:

**Table:** 

1	Left P5 basis with 1 longitudinal hyaline lamella on inner margin	***T. megahyaline***
–	Left P5 basis without hyaline lamella on inner margin	**2**
2	Left P5Exp with a uni-lobed inner margin	**3**
–	Left P5Exp with a bi-lobed inner margin	**8**
3	Inner margin of left P5Exp with uniform serration	**4**
–	Inner margin of left P5Exp with mixed-size serrations (large and small teeth)	**5**
4	Right P5 basis with 1 longitudinal hyaline lamella on inner margin; shape of right P5Exp-2 is rectangular	***T. kampucheaensis* sp. nov.**
–	Right P5 basis with 2 processes and 1 hyaline lamella on inner margin; shape of right P5Exp-2 is trapezoidal (proximal part narrower than distal part)	***T. ruttneri***
5	Right P5 basis with 3 processes on inner margin; left P5Exp with a series of denticles, remarkably large and getting larger halfway along inner margin	***T. foresti***
–	Right P5 basis with 2 processes on inner margin	**6**
6	Right P5 basis without hyaline lamella on inner margin; left P5Exp with a series of denticles, largest denticles in middle of inner margin	***T. oryzanus***
–	Right P5 basis with 1 hyaline lamella on inner margin	**7**
7	Shape of right P5Exp-2 is trapezoidal with a triangular-shaped hyaline structure located near base of principal lateral spine	***T. hebereri***
–	Shape of right P5Exp-2 is rhomboidal with a triangular-shaped hyaline structure located at middle of its segment	***T. pedecrassum***
8	Inner margin of left P5Exp with uniform serration	**9**
–	Inner margin of left P5Exp with mixed-size serrations	**13**
9	Posterior surface of right P5Exp-2 with a distinct longitudinal-shaped process located at middle near outer margin	***T. longiprocessus***
–	Posterior surface of right P5Exp-2 with a semicircular or triangular process located near base of principal lateral spine	**10**
10	Posterior surface of right P5Exp-2 with a semicircular knob located near base of principal lateral spine	**11**
–	Posterior surface of right P5Exp-2 with a triangular process located near base of principal lateral spine	**12**
11	Spinous process on antepenultimate segment of right antennule ~ 0.5× as long as segment 21; distal outer margin of right P5Exp-1 with a sharp spinous process	***T. doriai***
–	Spinous process on antepenultimate segment of right antennule ~ 0.75× as long as segment 21; distal outer margin of right P5Exp-1 with a blunt spinous process	***T. gigantoviger***
12	Spinous process on antepenultimate segment of right antennule longer than segment 21	***T. lanaonus***
–	Spinous process on antepenultimate segment of right antennule ~ 0.75 or 1.0× as long as segment 21	***T. vicinus***
13	Shape of right P5Exp-2 is cylindrical with a slender, long spine (~ 0.75× as long as length of principal lateral spine) located at proximal third of outer margin	***T. lannaensis* sp. nov.**
–	Shape of right P5Exp-2 is trapezoidal without a slender spine located at proximal third of outer margin	**14**
14	Inner margin of left P5Exp with mixed-size serrations, proximal lobe with uniform large denticles, distal lobe with small uniform denticles; spinous process on antepenultimate segment of right antennule shorter than segment 21	***T. vandouwei***
–	Inner margin of left P5Exp with mixed-size serrations, proximal lobe with distinctly larger denticles than those on distal lobe; spinous process on antepenultimate segment of right antennule longer than segment 21	***T. australis***

Females:

**Table:** 

1	Genital double-somite asymmetrical	**2**
–	Genital double-somite symmetrical	**11**
2	Caudal rami without outer setules	**3**
–	Caudal rami with outer setules	**4**
3	Fifth pediger with mid-dorsal roundish hump; right proximal margin of genital double-somite dilated into a round lobe, distal margin expanded into a large lobe	***T. hebereri***
–	Fifth pediger without mid-dorsal roundish hump; right proximal margin of genital double-somite not dilated, distal margin slightly expanded	***T. foresti***
4	Proximal and distal right margins of genital double-somite expanded	**5**
–	Only proximal or distal right margin of genital double-somite expanded	**7**
5	Right distal corner of genital double-somite swollen into a large triangular lobe	***T. pedecrassum***
–	Right distal corner of genital double-somite slightly swollen into a small round lobe	**6**
6	Left P5 coxal spine the same size as right spine	***T. australis***
–	Left P5 coxal spine much larger than right spine	***T. lannaensis* sp. nov.**
7	Only right proximal region of genital double-somite expanded; distal region not expanded into a lobe	**8**
–	Only right distal region of genital double-somite expanded; proximal region not expanded into a lobe	**9**
8	Right proximal 1/3 region of genital double-somite expanded into a round lobe	***T. longiprocessus***
–	Right proximal 2/3 region of genital double-somite slightly expanded	***T. kampucheaensis* sp. nov.**
9	P5Enp as long as Exp-1	***T. lanaonus***
–	P5Enp ~ 3/4 as long as Exp-1	**10**
10	P5 coxal spine long, ~ 1/2 as long as length of basis; fifth pediger with symmetrical wings	***T. vandouwei***
–	P5 coxal spine short, ~ 1/4 as long as length of basis; fifth pediger with asymmetrical wings	***T. ruttneri***
11	Both sides of genital double-somite not dilated subproximally	***T. gigantoviger***
–	Both sides of genital double-somite dilated subproximally	**12**
12	P5Exp-3 separate from Exp-2; outer margin of P5 end claw without spinules	***T. doriai***
–	P5Exp-3 fused with Exp-2	**13**
13	P5 coxal spine with a large lobe proximally and a triangular pointed spine distally	***T. oryzanus***
–	P5 coxal spine small, without lobe proximally	**14**
14	P5Enp with 2 rows of apical spinules	***T. megahyaline***
–	P5Enp with 1 row of apical spinules	***T. vicinus***

## ﻿Discussion

### ﻿Differential diagnosis of the two new species

According to our observations and review of literature (Kiefer 1982; Defaye 2002; Sanoamuang 2002; Ambedkar and Elia 2014; Saetang et al. 2020), members of the genus *Tropodiaptomus* can be distinguished from other genera by the following characteristics. In the males, (1) the second and third urosomites are usually without ventral hairs; (2) the ventral surface of the right caudal ramus lacks prominent structures; (3) the spinous process on the antepenultimate segment of the right antennule is slender, smooth, and slightly bent distally; (4) the Exp of the left P5 is one-segmented (Exp-1 and Exp-2 are fused flattened, and displays a serrated inner margin in posterior view, forming the ‘saw-like’ or ‘spatula-like’ shape, two central hairy pads; and (5) the apex of the Exp features a ‘finger-and-thumb’ combination, is ornamented with a spinulate seta. In the females, each P5Enp has two strong, unequal spiniform apical setae. The P2 Enp-2 in both sexes has a Schmeil’s organ on its posterior surface.

The two new species, *T. lannaensis* sp. nov. and *T. kampucheaensis* sp. nov., have the typical characteristics of *Tropodiaptomus* as described by Kiefer (1982) and summarized in the preceding paragraph. Among their Asian relatives, *T. lannaensis* sp. nov. resembles *T. ruttneri* in appearance. This similarity is particularly evident in the morphology of the spines on the grasping antennules, the male left P5Exp, and the male right P5Exp-2. However, several morphological characters distinguish *T. lannaensis* sp. nov. from *T. ruttneri* (Table 7). For instance, in males of *T. lannaensis* sp. nov.; the right P5 basis has two hyaline knobs on the inner margin proximally, instead of two hyaline knobs plus one hyaline lamella distally; the right P5Exp-2 is cylindrical, rather than trapezoidal; the right P5Exp-2 has a slender accessory lateral spine on the proximal third of the outer margin, in contrast to a small triangular spine located in the center of the posterior surface; and the distal inner margin of the left P5Exp is two-lobed with large denticles and small denticles near the distal end, instead of one-lobed with uniform denticles. The female characters in *T. lannaensis* sp. nov. differ from those in *T. ruttneri* in the following features: (1) the spines on the left and right P5 coxae are asymmetrical, with the spine on the right side larger and longer than that on the left side, whereas in *T. ruttneri*, the spines are symmetrical and equal in size on both sides; and (2) the sensory seta on the outer margin of the P5 basis is very short, reaching less than 1/4 of the Exp-1.

**Table 7.** Comparison of morphological characters of *Tropodiaptomus lannaensis* sp. nov. and *T. ruttneri*.

Characters	*Tropodiaptomus lannaensis* sp. nov.	*Tropodiaptomus ruttneri*
**Male**		
Total body length (excluding caudal setae)	1.1–1.2 mm	1.4–1.5 mm
Ornamentation on inner margin of right P5 basis	One semicircular knob and one triangular knob	One triangular knob, one semicircular knob and one longitudinal hyaline lamella
Shape of right P5Exp-2	cylindrical	trapezoidal
Supplementary projection on right P5Exp-2	A slender, long spine located at proximal third of outer margin	A small triangular spine located in the center of posterior surface
Distal inner margin of left P5Exp	Two-lobed, large denticles, small denticles near distal end	One-lobed, uniform denticles
**Female**		
Total body length (excluding caudal setae)	1.2–1.4 mm	1.5–1.6 mm
Spines on left and right P5 coxa	Asymmetrical, spine on left side larger and longer than right	Symmetrical, spines on both sides equal in size
Size of sensory seta on outer margin of P5 basis	Very short, reaching less than 1/4 of Exp-1	Short, reaching 1/4 of Exp-1

*Tropodiaptomus kampucheaensis* sp. nov. is similar to *T. doriai*, as evidenced by the following male characteristics: (1) a slender hyaline lamella on the inner margin of the right P5 basis; (2) the hyaline lamella on the right P5Exp-2; and (3) the ornamentation of the grasping antennules, especially the presence of a robust and spinous process on the 13^th^ segment. Nevertheless, *T. kampucheaensis* sp. nov. can be differentiated from *T. doriai* by several distinct male morphological features (Table 8): (1) the right P5 basis with one hyaline lamella in the new species, whereas two hyaline lamellae are present in *T. doriai*; (2) the right P5Enp is as long as the right Exp-1 in the new species, whereas it is longer than the right Exp-1 in *T. doriai*; (3) the right P5Exp-2 lacks an accessory lateral spine in the new species, whereas *T. doriai* has a small spine inserted distally; (4) the inner margin of the left P5Exp has one lobe in the new species, compared to two lobes in *T. doriai*; and (5) the left P5Enp is approximately half the length of the left Exp in the new species, whereas it is ~ 3/4 the length in *T. doriai*. In addition, the new species has an asymmetrical genital double-somite, while that of *T. doriai* is symmetrical. Furthermore, the outer margin of the P5Exp-2 claw has spinules, but it has no spinules in *T. doriai*.

**Table 8.** Comparison of morphological characters of *Tropodiaptomus kampucheaensis* sp. nov. and *T. doriai*.

Characters	*Tropodiaptomus kampucheaensis* sp. nov.	*Tropodiaptomus doriai*
**Male**		
Total body length (excluding caudal setae)	0.94–0.95 mm	1.10–1.50 mm
Ornamentation on inner margin of right P5 basis	one longitudinal hyaline lamella	two hyaline lamellae
Length of right P5Enp	as long as right Exp-1	longer than right Exp-1
Accessory lateral spine on right P5Exp-2	absent	one small spine located near base of principal lateral spine
Distal inner margin of left P5Exp	one lobed, uniform denticles	two lobed, uniform denticles
Length of left P5Enp	~1/2 length of left Exp	~3/4 length of left Exp
**Female**		
Total body length (excluding caudal setae)	1.53–1.55 mm	1.10–1.24 mm
Genital-double somite	asymmetrical (right margin expanded sub-proximally)	symmetrical
Outer margin of P5Exp-2 claw	spinules	unornamented

In addition to its distinction from *T. doriai*, *T. kampucheaensis* sp. nov. is further characterized by a unique combination of features that differentiate it from other congeners. In the male P5, the right basis has one hyaline lamella; the right Exp-2 has a small hyaline lamella near the base of the principal lateral spine; and the inner margin of the left Exp, forming a saw-like structure, is single-lobed with uniform minute serrations. In both sexes, the basis of P3 and P4 has an outer seta, and the P4 coxa has a distinctively long inner seta (Fig. 14D).

### ﻿Biogeography of *Tropodiaptomus* species in Southeast Asia

The distributional records of 16 species of *Tropodiaptomus* in Southeast Asia, including one unnamed taxon (*Tropodiaptomus* sp.) from Vietnam (Boonmak and Sanoamuang 2022: Fig. 2E), and their habitat types are presented in Table 9. Thailand is the most species-rich country for the genus, with ten species recorded so far (Sanoamuang and Dabseepai 2021; Saetang et al. 2020, 2022; Saetang and Maiphae 2023). This number includes a doubtful record of *T. doriai* in Thailand by Daday (1906), which requires a reconfirmation of its occurrence since we did not find this species in our extensive collection during the past 30 years (Sanoamuang and Dabseepai 2021). The species richness of *Tropodiaptomus* in Thailand is comparable to that of India, where it has nine recorded species, including two doubtful taxa (Ambedkar and Elia 2014). Indonesia has five species (Alekseev et al. 2013). The Philippines (Lai et al. 1979; Lopez et al. 2017) and Vietnam (Defaye 2002; Tran et al. 2016; Boonmak and Sanoamuang 2022) each have four species. Three species have been reported in Malaysia and Singapore (Lim and Lai 2014) and in Cambodia (Chaicharoen and Sanoamuang 2022). In contrast, only one species has been found in Laos (Sivongxay 2005).

**Table 9.** Distributions of the species in the genus *Tropodiaptomus* in Southeast Asia. Abbreviations that are used in the table: Per = permanent water, Tem = temporary water, Ca = Cambodia, In = Indonesia, La = Laos, Ma = Malaysia, Ph = Philippines, Si = Singapore, Th = Thailand, Vi = Vietnam. Symbols that are used in the table: + = present in the habitats, ✓ = present in the countries, and ? = doubtful record. No data are available for Myanmar and Brunei, so they are not included in this table.

*Tropodiaptomus* species	Habitats		Occurrence in Southeast Asia							Occurrence in other countries
	Per	Tem	Ca	In	La	Ma & Si	Ph	Th	Vi	
*T. australis* Kiefer, 1936	+	+		✓			✓			Australia, South Africa, China, India, Sri Lanka
*T. doriai* (Richard, 1894)	+			✓				?		India, Sri Lanka
*T. foresti* Defaye, 2002		+							✓	–
*T. gigantoviger* Brehm, 1933	+						✓			–
*T. hebereri* (Kiefer, 1930)	+	+		✓		✓		✓		China, India
*T. kampucheaensis* sp. nov.		+	✓							–
*T. lanaonus* Kiefer, 1982							✓	✓		–
*T. lannaensis* sp. nov.		+						✓		–
*T. longiprocessus* Saetang & Maiphae, 2023	+							✓		–
*T. megahyaline* Saetang, Sanoamuang & Maiphae, 2021		+						✓		–
*T. oryzanus* Kiefer, 1937		+	✓					✓	✓	China, Japan, Korea, Taiwan
*T. pedecrassum* Saetang & Maiphae, 2023	+							✓		–
*T. ruttneri* (Brehm, 1923)	+					✓		✓		China
*T. vandouwei* (Früchtl, 1924)	+			✓						–
*T. vicinus* (Kiefer, 1930)	+	+	✓	✓	✓	✓	✓	✓	✓	India
*Tropodiaptomus* sp. in Boonmak and Sanoamuang (2022)		+							✓	–
**Total**	**9**	**9**	**3**	**5**	**1**	**3**	**4**	**10**	**4**	

Of the 16 Southeast Asian species of *Tropodiaptomus*, most are restricted to this region. However, only six species have been recorded outside the area, including *T. australis*, *T. doriai*, *T. hebereri*, *T. oryzanus*, *T. ruttneri*, and *T. vicinus*. The distribution of these six species extends from Southeast Asia to South and East Asia. *Tropodiaptomus australis* has the widest distribution, occurring from Indonesia and the Philippines to Australia, South Africa, China, India, and Sri Lanka. *Tropodiaptomus oryzanus* also has a wide distribution, from Thailand, Cambodia, and Vietnam to China, Japan, Korea, and Taiwan. *Tropodiaptomus vicinus* is the most common species in Southeast Asia, with records from Thailand, Cambodia, Laos, Vietnam, Malaysia, Singapore, Indonesia, the Philippines, and India (Table 9). *Tropodiaptomus lanaonus* has been found only in Southeast Asia: the Philippines and Thailand. Four newly described species are probably endemic to Thailand: *T. longiprocessus*, *T. megahyaline*, *T. lannaensis* sp. nov., and *T. pedecrassum*. Two species (*T. foresti* and *Tropodiaptomus* sp.) are endemic to Vietnam. *Tropodiaptomus gigantoviger* is endemic to the Philippines, and *T. vandouwei* to Indonesia.

Regarding their habitat occurrence, nine species of *Tropodiaptomus* have so far been found in permanent-water habitats and the other nine in temporary-water habitats (Table 9). Three species (*T. australis*, *T. hebereri*, and *T. vicinus*) have been reported in both permanent and temporary-water habitats. Seven species have been recorded only in temporary-water habitats, including the five recently recorded taxa: *T. foresti*, *T. megahyaline*, *T. kampucheaensis* sp. nov., *T. lannaensis* sp. nov., and *Tropodiaptomus* sp. from Vietnam. These taxa may have been previously overlooked in temporary-water habitats. Species with the ability to adapt to both temporary and permanent habitats (e.g., *T. australis*, *T. hebereri*, and *T. vicinus*) may have primarily wider distributions than those restricted to one type (Collinson et al. 1995), although it is not the case in *T. oryzanus*, which occurs only in temporary-water habitats but has a wide distribution. In conclusion, both temporary and permanent habitats are necessary for maintaining biodiversity, and the ability of species to utilize both types of habitats can significantly impact their distribution patterns.

Among the diaptomid genera recorded in Thailand, *Tropodiaptomus* and *Mongolodiaptomus* Kiefer, 1937, are the most species-rich, with ten species identified in each (Sanoamuang and Koompoot 2024; this study). However, the number of individuals observed in each sampling site of *Tropodiaptomus* species was consistently low. Frequently, we found just a limited number of specimens, sometimes even a single individual, at each sampling site. Therefore, we believe that conducting intensive collections throughout Southeast Asia could lead to the discovery of more undescribed species of this genus.

## Supplementary Material

XML Treatment for Tropodiaptomus lannaensis

XML Treatment for Tropodiaptomus kampucheaensis

## ﻿Additional information

### Use of AI

No use of AI was reported.

### Author ORCIDs

Kamonwan Koompoot https://orcid.org/0009-0005-2116-5191

Laorsri Sanoamuang https://orcid.org/0000-0003-0377-1225

### Data availability

All of the data that support the findings of this study are available in the main text.
